# Microcirculatory alterations are associated with pulmonary dead-space fraction in moderate and severe ards

**DOI:** 10.1186/2197-425X-3-S1-A453

**Published:** 2015-10-01

**Authors:** GA Ospina-Tascón, DF Bautista-Rincón, HJ Madriñán, JD Valencia, WF Bermúdez, A Bruhn, G Hernández, CE Salas, D De Backer

**Affiliations:** Department of Intensive Care Medicine, Fundación Valle del Lili - Universidad ICESI, Cali, Colombia; Departamento de Medicina Intensiva, Pontificia Universidad Catolica de Chile, Facultad de Medicina, Santiago, Chile; Intensive Care Medicine Department, CHIREC Hospitals, Université Libre de Bruxelles, Brussels, Belgium

## Introduction

Shunt-induced hypoxemia is considered the primary pathophysiological abnormality and main diagnostic criteria of acute respiratory distress syndrome (ARDS). However, increases in dead-space ventilation (VD/VT) can also contribute to gas exchange alterations in ARDS. Systemic microcirculatory alterations described during inflammatory conditions are characterized by perfusion heterogeneity and theoretically pulmonary microcirculatory heterogeneity could lead to imbalance pulmonary ventilation/perfusion relationship. Thus, we hypothesized that systemic microvascular alterations could reflect increased VD/VT in ARDS.

## Objectives

To evaluate the prognostic value and the relationship between the VD/VT and microvascular blood flow derangements during the early stages of moderate and severe ARDS.

## Methods

Prospective observational in an 80-bed mixed ICU. Patients meeting the Berlin’s ARDS criteria and ventilated by at least 12 hours were pre-selected. Those with a PaO_2_/FiO_2_ < 200 after a 1-hour trial with a FiO_2_ of 1.0, PEEP ≥10 cmH_2_O and VT of 6-8 ml/kg of predicted body weight were finally included. The Bohr’s approach was used to measure the VD/VT by means of volumetric capnography. After a recruitment maneuver, PEEP was set targeting the highest respiratory compliance and the lowest VD/VT. After 60-minutes of stabilization, sublingual microcirculatory images were acquired using a Sidestream-dark Field device (SDF) for ulterior semi-quantitative analysis. Continuous microvascular flows were considered normal while sluggish, intermittent and stopped flows were considered abnormal and a cutoff of 20 µm was used to classify microvessels as large or small.

## Results

**Figure 1:**
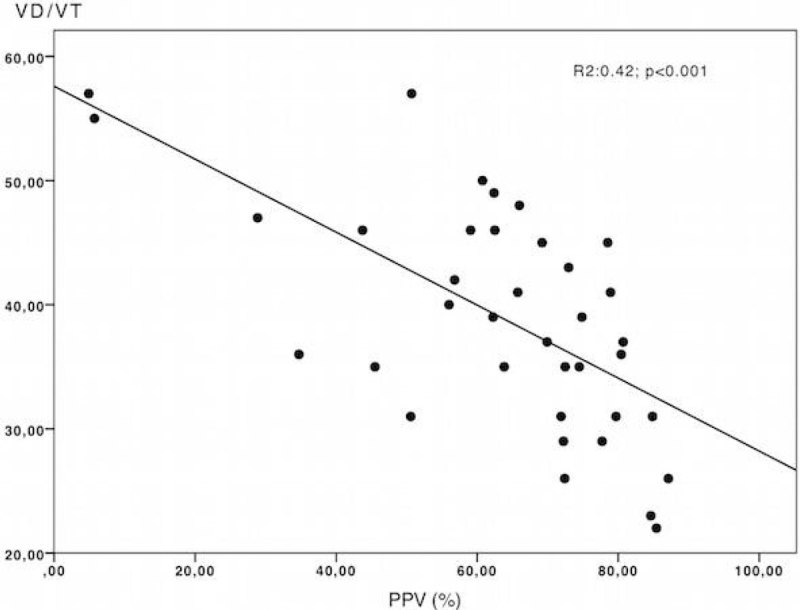
**VD/VT vs. PPV.**

A total of 38 patients with moderate and severe ARDS were included. Respiratory mechanics, blood gases analysis and VD/VT for survivors and non-survivors are presented in the table 1. A univariate analysis demonstrated that functional capillary density, the percentage of small vessels perfused (PPV), and VD/VT were significantly different between survivors and non-survivors at day-90. However, in the multivariate analysis only the VD/VT remained associated with mortality at day-90 (coefficient: 1.253 95%CI: 1.018 to 1.542, p = 0.033). An inverse relationship was also observed between the PPV and the VD/VT (R^2^:0.42, p < 0.001)(Figure 1). Meanwhile, a linear regression model demonstrated that PPV was significantly related to VD/VT changes (coefficient: -0.288 95%CI: -0.429 to -0.147, p < 0.001) while other respiratory mechanics and oxygenation parameters did not. When we explored the population equipped by a pulmonary artery catheter, we found that PPV and cardiac index were related to VD/VT.

**Table 1:** Respiratory mechanics, blood gases and VD/VT.

	All	Survivors	Non-Survivors	p
VT (ml/kg)	6.5 (6.2 - 6.9)	6.5 (6.2 - 6.9)	6.6 (6.1 - 7.5)	0.47
RR	24 (20 - 26)	24 (22 - 27)	24 (20 - 26)	0.44
PEEP	12 (10 - 14)	12 (10 - 14)	14 (10 - 15)	0.19
Pplat-RS	25 (24 - 30)	28 (24 - 32)	31 (28 - 34)	0.09
VT/C-RS	14 (17 - 19)	16 (14 - 18)	17 (14 - 21)	0.44
PaO2/FiO2	120.6 (89.6 - 157.1)	122.5 (92.3 - 178.4)	116.1 (83.6 - 138.8)	0.11
PaCO2	48.5 (37.9 - 56.7)	41.2 (34.4 - 55.1)	51.8 (43.7 - 61.8)	0.05
pH	7.27 (7.21 - 7.31)	7.30 (7.25 - 7.33)	7.23 (7.14 - 7.29)	0.01
VD/VT	39 (31 - 46)	35 (29 - 39)	46 (36 - 49)	0.001

## Conclusions

Increased dead-space ventilation seems to be superior to respiratory mechanics and oxygenation parameters to predict outcomes in moderate and severe ARDS. Alterations in the microvascular blood flow might influence early increases in the dead-space ventilation.

